# A controlled trial of protein enrichment of meal replacements for weight reduction with retention of lean body mass

**DOI:** 10.1186/1475-2891-7-23

**Published:** 2008-08-27

**Authors:** Leo Treyzon, Steve Chen, Kurt Hong, Eric Yan, Catherine L Carpenter, Gail Thames, Susan Bowerman, He-Jing Wang, Robert Elashoff, Zhaoping Li

**Affiliations:** 1 UCLA Center for Human Nutrition, Department of Medicine, David Geffen School of Medicine at UCLA, Los Angeles, USA; 2 Department of Biomathematics, David Geffen School of Medicine at UCLA, Los Angeles, USA

## Abstract

**Background:**

While high protein diets have been shown to improve satiety and retention of lean body mass (LBM), this study was designed to determine effects of a protein-enriched meal replacement (MR) on weight loss and LBM retention by comparison to an isocaloric carbohydrate-enriched MR within customized diet plans utilizing MR to achieve high protein or standard protein intakes.

**Methods:**

Single blind, placebo-controlled, randomized outpatient weight loss trial in 100 obese men and women comparing two isocaloric meal plans utilizing a standard MR to which was added supplementary protein or carbohydrate powder. MR was used twice daily (one meal, one snack). One additional meal was included in the meal plan designed to achieve individualized protein intakes of either 1) 2.2 g protein/kg of LBM per day [high protein diet (HP)] or 2) 1.1 g protein/kg LBM/day standard protein diet (SP). LBM was determined using bioelectrical impedance analysis (BIA). Body weight, body composition, and lipid profiles were measured at baseline and 12 weeks.

**Results:**

Eighty-five subjects completed the study. Both HP and SP MR were well tolerated, with no adverse effects. There were no differences in weight loss at 12 weeks (-4.19 ± 0.5 kg for HP group and -3.72 ± 0.7 kg for SP group, p > 0.1). Subjects in the HP group lost significantly more fat weight than the SP group (HP = -1.65 ± 0.63 kg; SP = -0.64 ± 0.79 kg, P = 0.05) as estimated by BIA. There were no significant differences in lipids nor fasting blood glucose between groups, but within the HP group a significant decrease in cholesterol and LDL cholesterol was noted at 12 weeks. This was not seen in the SP group.

**Conclusion:**

Higher protein MR within a higher protein diet resulted in similar overall weight loss as the standard protein MR plan over 12 weeks. However, there was significantly more fat loss in the HP group but no significant difference in lean body mass. In this trial, subject compliance with both the standard and protein-enriched MR strategy for weight loss may have obscured any effect of increased protein on weight loss demonstrated in prior weight loss studies using whole food diets.

## Background

Meal replacement shakes represent an important strategy in combating the worldwide epidemic of obesity due to their simplicity and convenience [1]. Meal replacement shakes have been studied extensively for both medical and public health efforts to combat obesity [2-4].

A number of studies have suggested that protein is the most important macronutrient mediating satiety and leads to increased weight loss with retention of lean body mass. Single meals with increased protein to carbohydrate ratios have also been shown to increase satiety and decrease food intake [5,6], resulting in both improved weight loss and improved maintenance of weight loss [7-9]. Meal replacement simplifies the weight loss regimens by replacing one or two meals a day with a product of defined nutrient and calorie content. MR leads to increased weight losses over twelve weeks compared to simply restricting favorite food intakes, and weight losses have been maintained for up to five years using MR [10]

An increase in dietary protein content has been proposed to be effective for body weight regulation through effects on satiety, thermogenesis and substrate partitioning. Protein has specific effects on satiety hormones, including PYY 3–36 [11]. When protein replaces carbohydrate within a low-fat diet, reduced insulinemic and glycemic responses have been observed resulting in increased fat oxidation [12]

The present study was designed to test the hypothesis that simply increasing the protein content of a meal replacement (MR) within a high protein diet without the knowledge of the participant would result in increased weight loss and improved retention of lean body mass in the absence of a resistance exercise program by comparison to standard MR within a standard protein diet. To test the hypothesis, a soy and whey protein powder was used to enrich a standard MR shake in one arm compared to a carbohydrate "placebo" powder added to the same MR shake in the other arm. This novel approach has not been tested previously to our knowledge. To minimize variations based on body composition, the diets were also adjusted so that each subject was instructed to follow a diet which provided either 2.2 gm/kg lean body mass protein in the high protein (HP) group or 1.1 gm protein/kg lean body mass in the standard protein (SP) group. Patients received dietary instruction at baseline, and met with the dietitian at weeks 2, 4 and 8 to assess general compliance and to provide additional supplies of the MR products. Therefore, this study examines the effectiveness of protein enrichment of MR in a realistic outpatient setting on weight loss and retention of lean body mass.

## Methods

Subjects were recruited by public advertisement. Subjects over 30 years of age with a body mass index (BMI) between 27 to 40 kg/m^2^, and in good health by history, physical examination, and basic laboratory screening (complete blood count, serum chemistries, liver panel, and lipid panel) were selected for study. Subjects with type 2 diabetes or glucose intolerance were excluded as were individuals who regularly drank more than one alcoholic beverage daily,

One hundred men and women who met the selection criteria were randomly assigned to either the HP or SP treatment. This was a single-blinded study. The protein powder jars were labeled as either A or B, depending on their protein content. Subjects were randomized in a 1:1 manner to either HP or SP diet for 12 weeks using a computerized random proportion model. Diet plans were individualized per subject. Caloric intake to achieve weight loss was based on a 500 Kcal deficit of the participants' estimated resting metabolic rate as determined by body composition analysis by bioelectrical impedance.

Participants in the HP group received a diet plan that provided 2.2 grams of protein per kg of LBM while the diet for the SP group provided 1.1 grams of protein per kg of LBM. The meal energy macronutrient composition in the HP group was approximately 30% protein, 30% fat, and 40% carbohydrate. The macronutrient composition in the SP diet was approximately 15% protein, 30% fat, and 55% carbohydrate. Both groups received the same isocaloric MR (Formula 1, Herbalife Intl., Los Angeles) with either a protein supplement for the HP group (Performance Protein Powder, Herbalife Intl., Los Angeles) or with a similar tasting carbohydrate placebo for SP group. Two MR and two meals were eaten daily.

Instructions were provided for preparation of the MR and subjects were advised to consume one MR as a meal and the other as snack. All subjects were given individualized menu plans that incorporated the two MR (one meal and one snack) and included two all-food meals. All participants met individually with a registered dietitian at baseline for dietary instruction, and at 2, 4, and 8 weeks to assess compliance.

Participants were weighed and protein powder meal replacement products were dispensed at each visit to ensure compliance. Subjects were given general advice for increasing their activity level with a goal of 30 minutes of aerobic exercise per day, but no heavy resistance exercise.

### Body weight and composition

Subjects were weighed at each visit (Detecto-Medic; Deteco-Scales; Brooklyn, NY) while wearing no shoes and after an overnight fast. Height was measured with a stadiometer (Detecto-Medic; Deteco-Scales; Brooklyn, NY) at week 0. BMI was calculated as weight (kg)/height squared (m). Body composition was determined by bioelectrical impedance analysis (BIA) (310e Bioimpedance analyzer; Biodynamics; Seattle, WA) and was performed at 0 and 12 weeks.

### Biochemistry

Fasting blood samples were collected at weeks 0, 4, 8, and 12 for measurement of lipid profiles, blood glucose and liver function tests.

### Statistical analysis

Weight loss was the primary outcome and the data were analyzed according to intention to treat allocation utilizing SAS version 9 (Cary, North Carolina) in the Department of Biostatistics.

Patient characteristics and baseline measurements of the two study groups were compared using t-test (for numerical variables) or Chi-square test (for categorical variables) to evaluate quality of the randomization.

Standard t-tests were used to compare weight losses between the two arms. In addition, to assess weight loss within each treatment arm, paired t-tests were conducted comparing baseline and 12 week weight for each subject. All data except baseline characteristics are presented as means +/- standard error of the mean (SEM). A univariate analysis of variance was used to assess differences between treatment and outcome variables. Since the distributions of change in fat weight and percentage change in fat weight were not normal, signed rank test was used for testing change from baseline within each group. The Wilcoxon rank sum test was used for comparing the change between the two groups. Multivariate analysis was performed to compare the difference between the two diet groups using general linear model. Square root transformation was applied before the multivariate analysis was performed.

## Results

100 obese men and women were randomly assigned to either a HP or SP MR diet plan. Fifteen subjects withdrew from the study within the first week after randomization due to noncompliance with the meal plan (6 in the HP group and 9 in the SP group). All other subjects completed the 12-week study. Subject characteristics in the two treatment arms at baseline were not significantly different (Table 1). Mean age was 49.4 ± 1.1 years. Mean BMI at baseline was 33.8 ± 0.53 for HP group and 32.6 ± 0.58 kg/m2 for SP group.

**Table 1:** Patient characteristics at baseline

	HP (N = 45)	SP (N = 42)	Total (N = 87)	HP vs. SP
Gender				
F	34 (76%)	27 (64%)	61 (70%)	NS
M	11 (24%)	15 (36%)	26 (30%)	
Age				
Mean ± SE	49.2 ± 1.8	49.7 ± 1.4	49.4 ± 1.1	NS
Median, range	47.0, 28–69	49.5, 30–65	49.0, 28–69	
Race				
Asian	4 (9%)	1 (2%)	5 (6%)	NS
Black	9 (20%)	7 (17%)	16 (18%)	
Caucasian	25 (55%)	30 (72%)	55 (63%)	
Hispanic	4 (9%)	2 (5%)	6 (7%)	
Other	0	1 (2%)	1 (1%)	
Unknown	3(7%)	1 (2%)	4(5%)	

### Weight loss

**Figure 1:**
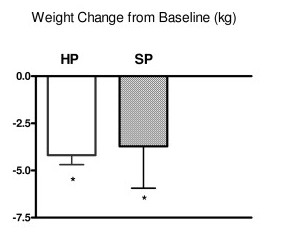
Weight change from baseline at 12 weeks. * p < 0.05 compared with base line body weight. Blank bar represents the high protein group, the shaded bar represents the standard protein group.

Subjects were weighed at baseline, and at 2, 4, 8 and 12 weeks. Baseline body weight was not significantly different between these two groups. Both groups lost significant amount of weight at 12 weeks (-4.19 ± 0.5 kg for HP group and -3.72 ± 0.7 kg for SP group, p < 0.0001 for both groups). (Figure 1) After controlling for baseline weight, gender, and time period, there was no significant difference between the two treatment groups. For both dietary groups, BMI was significantly lower at 12 weeks (HP = -1.50 ± 0.58; SP = -1.13 ± 0.24). There were no significant differences in BMI changes between the two dietary groups (Table 2).

**Table 2:** Change of BMI, waist circumference, fat mass, and fat fee mass at 12 weeks

	BMI ((kg/m2)		Waist Circumference (cm)		Fat Mass (kg)		Fat Free Mass (kg)	
	HP	SP	HP	SP	HP	SP	HP	SP

Baseline	33.77 ± 0.53	32.66 ± 0.58	104.2 ± 1.8	101.7 ± 2.0	35.2 ± 1.0	32.3 ± 1.3	58.3 ± 1.6	60.0 ± 1.9
12 weeks	32.13 ± 0.54	31.11 ± 0.56	98.8 ± 1.6	97.3 ± 2.0	33.6 ± 1.2*	31.7 ± 1.0	55.6 ± 1.4	55.9 ± 1.7

*p < 0.0001 compare 12 weeks vs. baseline

### Waist circumference

Change in waist circumference (cm) at 12 weeks was significant in both treatment groups (HP = -6.7 ± 1.1; SP = -5.1 ± 0.8 p < 0.0001). No significant differences in change in waist circumference at any time period were observed between diets (table 2).

### Fat mass by BIA

**Figure 2:**
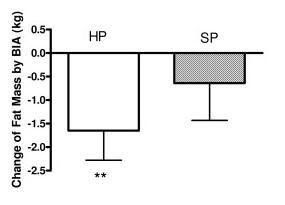
**Change of fat mass by BIA from baseline at 12 weeks.** ** p < 0.001 compared with base line fat mass. Blank bar represents the high protein group and the shaded bar represents the standard protein group.

Subjects in the HP group lost a significant amount of fat at 12 weeks (from 35.2 ± 1.0 kg to 33.6 ± 1.2 kg, p < 0.0001) but not the SP group (32.3 ± 1.3 kg to 31.7 ± 1.0 kg, p > 0.05). Subjects in the HP group lost significantly more fat weight than the SP group (HP = -1.65 ± 0.63; SP = -0.64 ± 0.79 kg p = 0.05) (Figure 2, table 2).

### Fat-free mass by BIA

At 12 weeks, the two dietary groups had significantly decreased lean body weight (kg) (HP = -2.78.1 ± 0.62; SP = -4.06 ± 1.74, p < 0.0001). No significant differences were observed between the dietary groups (table 2).

### Cholesterol, HDL, LDL, triglyceride, and glucose

At 12 weeks, there were significant reductions in cholesterol and LDL levels (mg/dL) for the HP group (cholesterol -13.2 ± 5.3, p < 0.05; LDL -7.47 ± 3.38, p < 0.05) but not for the SP group (cholesterol -7.02 ± 4.3 p > 0.05; -9.17 + 5.65, p > 0.05). The difference between the two groups was not significant. There were no significant changes from baseline, nor between dietary groups in serum HDL and triglyceride levels. Similarly, fasting blood glucose levels did not change significantly from baseline for either group at 12 weeks. (Table 3)

**Table 3:** Change of lipid profiles and blood glucose

	Cholesterol (mg/dL)		LDL (mg/dL)		HDL (mg/dL)		Triglycerides (mg/dL)		Glucose (mg/dL)	
	HP	SP	HP	SP	HP	SP	HP	SP	HP	SP

Baseline	199.82 ± 6.91	204.00 ± 6.07	118.16 ± 5.41	128.07 ± 5.69	54.45 ± 2.05	52.43 ± 1.69	122.16 ± 10.06	117.71 ± 8.44	89.25 ± 15.62	84.84 ± 11.40
12 weeks	186.69 ± 5.78*	195.49 ± 5.49	109.76 ± 4.68*	117.49 ± 5.65	55.04 ± 2.23	55.15 ± 2.75	107.44 ± 8.12	114.51 ± 9.92	85.35 ± 17.19	84.12 ± 12.90

*p < 0.05 compare 12 weeks vs. baseline

## Discussion

Protein-enriched meal replacements within a higher protein diet resulted in no greater overall weight loss than the standard protein MR plan over 12 weeks. In this trial, the amounts of weight lost were typical for meal replacement studies done previously [10]. However, the expected effects on increased weight loss resulting from a high protein diet were not seen in this study. There are two possible reasons for the observed similarities in overall weight loss. First, the subjects in the SP group may have eaten foods outside their recommended meal plans which increased protein intake enough to compensate for the difference in protein contents of the MR. Second, the use of MR may have been the major influence on the weight loss by simplifying their weight loss efforts so that the power of the MR intervention may have obscured the difference between the weight loss of subjects using protein-enriched MR shakes by comparison to standard MR [13]. The purpose of the study was to test the real world impact of simply enriching MR with more protein. Based on our results, it appears that compliance is a much more important factor in the MR regimen than protein content.

Protein enrichment of MR did appear to lead to increased retention of lean body mass based on bioelectrical impedance analysis. In this study, greater retention of lean body mass was suggested by the observation of increased fat loss at similar weight losses. Fat loss is determined by subtracting the lean body mass determined based on body water content from the total body weight at baseline compared to 12 weeks. The fat loss was significant both between groups and in individuals over time. Loss of lean mass was not different since the variability in fat free mass between subjects increased the variability of this measurement, reducing our power to see any difference. The observation we made at 12 weeks using bioelectrical impedance will require confirmation in longer-term studies where changes in body composition are more marked and in which additional methods for determining body composition are used. A recent meta-analysis of 87 short-term diet studies where protein and carbohydrate content was varied found that a protein intake of greater than 1.05 g/kg of actual body weight was associated with 0.6 kg additional fat-free mass retention compared with diets with protein intakes ≤1.05 g/kg [14]. Both meal plans in this study had protein greater than this cut point and the effects seen may be blunted by the relatively high protein in the SP group.

In future studies, it may also be desirable to combine protein enrichment of MR with resistance exercise to demonstrate significant differences in the retention of lean body mass during weight loss due to protein enrichment of MR. Evans and co-workers have shown that healthy free-living elderly men and women accommodate to the Recommended Dietary Allowance (RDA) for protein of 0.8 grams/kilogram/day with a continued decrease in urinary nitrogen excretion and reduced muscle mass. Increased dietary protein intake (up to 1.6 g protein/kg/day) may also enhance the hypertrophic response to resistance exercise that would enhance weight loss maintenance [15].

## Conclusion

In summary, both the HP and SP diets resulted in the expected weight loss typical of an MR diet plan at 12 weeks. Both diets were well tolerated, sustainable, and did not result in any adverse effects. While typical results for outpatient trials of MR were observed in both arms, greater compliance with the MR diet plan may be necessary to obtain an improved sense of the contribution of protein enrichment of MR to lean body mass retention during weight loss. Finally, future studies may be more successful if they include a comparison of standard MR and protein-enriched of MR weight reduction regimens combined with heavy resistance exercise to maintain or increase lean body mass.

## Competing interests

The authors declare that they have no competing interests.

## Authors' contributions

All authors read and approved the final manuscript. LT participated in the conduct of the study, the analysis of the data, and drafted the manuscript. SC participated in the conduct of the study. KH participated in the conduct of the study. EY participated in the conduct of the study. CLC participated in the study design and statistical analysis. GT participated in the study coordination. HW participated in the statistical analysis. RE participated in the statistical analysis. ZL conceived of the study, participated in its design and coordination, and drafted the manuscript.
